# Mechanical Testing of a Novel Fastening Device to Improve Scoliosis Bracing Biomechanics for Treating Adolescent Idiopathic Scoliosis

**DOI:** 10.1155/2018/7813960

**Published:** 2018-08-12

**Authors:** Chloe L. Chung, Derek M. Kelly, Jeffery R. Sawyer, Jack R. Steele, Terry S. Tate, Cody K. Bateman, Denis J. DiAngelo

**Affiliations:** ^1^BioRobotics Laboratory, Department of Orthopaedic Surgery and Biomedical Engineering, The University of Tennessee Health Science Center, Memphis, TN, USA; ^2^Campbell Clinic Orthopaedics and Le Bonheur Children's Hospital, Memphis, TN, USA; ^3^The Center for Orthotics and Prosthetics Inc., Memphis, TN, USA

## Abstract

Velcro fastening straps are commonly used to secure a scoliosis brace around the upper body and apply corrective forces to the spine. However, strap loosening and tension loss have been reported that reduce spinal correction and treatment efficacy. A novel fastening device, or controlled tension unit (CTU), was designed to overcome these limitations. A scoliosis analog model (SAM) was used to biomechanically compare the CTU fasteners and posterior Velcro straps on a conventional brace (CB) as well as on a modified brace (MB) that included a dynamic cantilever apical pad section. Brace configurations tested were (1) CB with posterior Velcro straps, (2) CB with posterior CTU fasteners, (3) MB with posterior Velcro straps, and (4) MB with posterior CTU fasteners. MB configurations were tested with 0 N, 35.6 N, and 71.2 N CTU fasteners applied across the apical pad flap. Three-dimensional forces and moments were measured at both ends of the SAM. The CTU fasteners provided the same corrective spinal loads as Velcro straps when tensioned to the same level on the CB configuration and can be used as an alternative fastening system. Dynamically loading the apical flap increased the distractive forces applied to the spine without affecting tension in the fastening straps.

## 1. Introduction

Scoliosis is a three-dimensional (3D) skeletal deformity of the spine consisting of axial rotation and lateral curvature (Cobb angle) [1]. An estimated 6 million people are affected in the United States alone. Adolescent idiopathic scoliosis (AIS) has an unknown cause and represents approximately 2–3% of the pediatric population [2]. It is estimated that out of the 600,000 annual patient visits, 30,000 [3] are considered moderate (with spinal curves less than 45°) and treated with a brace and 38,000 are considered severe or rapidly progressive (when the curve exceeds 50°) and treated with spinal fusion surgery [2]. These surgical treatments are very costly and usually increase health risks. As of 2012, the mean AIS spinal fusion hospital charges were $177,176 [4], while bracing treatment averaged $4000 per patient [5]. Recent clinical findings[6, 7] have shown bracing treatment to be 72% effective in preventing curve progression pass 50°, and more clinicians are now considering it as a treatment option. The primary function of scoliosis braces is to apply corrective forces to the spine that reduce and prevent progression of the spinal deformity [4, 8, 9].

Scoliosis braces can be classified as soft/flexible braces, semirigid braces, or hard/rigid Braces. Soft and semirigid braces provide limited torso stabilization with poor force corrective capacity and were proven to be less effective than hard/rigid braces [10]. Hard/rigid braces, such as the Boston Brace, Milwaukee Brace, Charleston Bending Brace, and Providence Brace, are the most frequently used braces [2] in the United States. These braces primarily consist of a rigid shell that fits over the patient's upper torso and pelvis anatomy.

The brace serves to maintain and, in some cases, reduce [9] the spinal curve to prevent progression of the deformity by applying corrective forces while being worn [11]. Many braces use a three-point pressure principle as the method of correction, which involves fixation above, below, and at the apex of the curve [12]. In Boston braces, foam pads are placed at specific locations within the brace to stabilize the anatomy and achieve correction of the lateral curve and malrotation [12]. These pads serve to stabilize the anatomy (trochanter pads) and apply corrective forces to the spine (lumbar pads, thoracic pads, and derotation pads). The magnitude and direction of these corrective forces applied by the brace to the spine remain unknown and are a common concern for clinicians and orthotists. In addition to pad sizing and placement, orthotists carry out other design alterations to custom fit the brace to the patient including the addition of a number of fastening devices, setting the tension of the fastening devices, and location and size of cut-out sections.

Fastening devices, like Velcro straps, are commonly used to tighten and secure the brace around the upper body by an orthotist using professional judgment and patient-reported comfort and are responsible for the majority of the corrective forces applied to the spine [13]. Typical strap tension settings are provided in Table 1 and vary between 20 N and 60 N [2, 3, 4]. However, Velcro strapping systems have been associated with strap loosening and tension loss following two or more weeks of daily brace wear [14], after various daily living activities [5, 14], or when lying down [4, 14–17]. The American Academy of Orthotists and Prosthetists reported that “loss of strap tension in scoliosis bracing could be a direct link to loss of in-orthosis correction” [18] and hence brace efficacy as well. Loss of strap tension decreases the corrective beneficial characteristics of the brace that negates the benefit of wearing the brace. In addition, besides reduced corrective forces and spinal correctional losses, braces have also been reported as being uncomfortable to wear, resulting in reduced brace wear time or complete abandonment [19, 20]. Although monitoring systems can be employed to determine if the user wears the brace the prescribed time [14, 21], active adjustment to correct the strap tension loss is not yet available. Currently, orthotists mark on the Velcro straps to indicate the prescribed level of strap tension and request the patients and parents to follow accordingly when wearing the brace. However, if the strap is placed short of this mark, the strap tension will be below the original prescribed value. The ongoing loosening of Velcro straps perpetuates the need for the strap monitoring/adjustment cycle [22]. To that end, there remains a need for a scoliosis brace fastening system that maintains the prescribed tension level set by the practitioner over the daily usage of the brace.

A modified fastening device was designed to overcome loss of strap tension associated with conventional Velcro straps during movements and increase the corrective forces (via strap tension) without compromising user comfort and treatment efficacy [2, 3, 5]. The device consisted of a constant force spring and custom enclosure that readily attached to the existing Velcro straps as an interface to attach the unit enclosure to the surface of the brace.

The first objective was to compare Velcro strap fasteners and the novel constant tension unit (CTU) fastening devices on a standard conventional brace (CB). An existing scoliosis analog model and robotic testing platform were used to analyze their biomechanical effect on maintenance of strap fastening tension as well as the amount of corrective brace force applied to the spine. The CB with no posterior fastening straps was also tested to remove the native stiffness contribution of the brace itself. The second objective was to evaluate the biomechanical effects of a modified brace (MB) on the corrective forces applied by the brace compared to a conventional brace configuration for the same scoliosis deformity.

The MB resembled a CB brace with the addition of a dynamizing cantilevered apical pad section. Four brace configurations were tested in the second objective: (1) CB with posterior Velcro straps, (2) CB with posterior CTU fasteners, (3) MB with posterior Velcro straps, and (4) MB with posterior CTU fasteners.

## 2. Materials and Methods

### 2.1. Controlled Tension Unit Device

**Figure 1:**
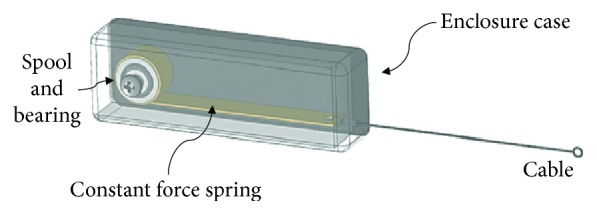
Schematic illustration of a controlled tension unit device consisting of a constant force spring, spool and bearing, cable, and enclosure case.

The controlled tension unit (CTU) device consisted of a constant force spring, enclosure case, and cable connectors (Figure 1). The constant-force spring within the CTU device consisted of a number of laminated springs (Century Spring model CF103). The original springs were made of material that is 0.005 inches thick and 0.312 inches wide, and they were rated for 1.030 lb ± 10%. After laminating the desired number of springs together around a low-friction spool, the laminated spring was mounted within the CTU housing. The number of springs laminated depended on the desired force output. The force output and load tolerance were validated using an in-line load cell during a controlled displacement test [23]. The CTU assemblies were determined to have a functional working length of 12.7 mm and a load tolerance of ±10% of the desired force output and were attached to the surface of the brace with Velcro strips.

### 2.2. Brace Configurations

**Figure 2:**
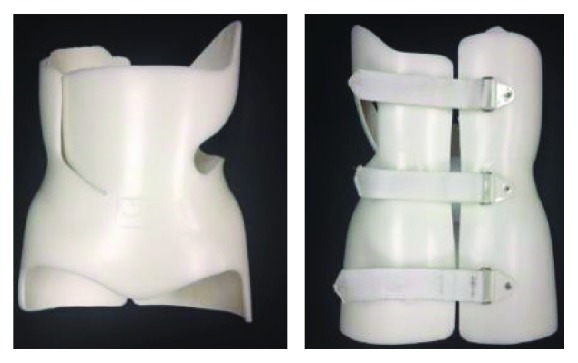
Conventional brace from Boston Brace International with three posterior fastening straps and an apical flap section.

**Figure 3:**
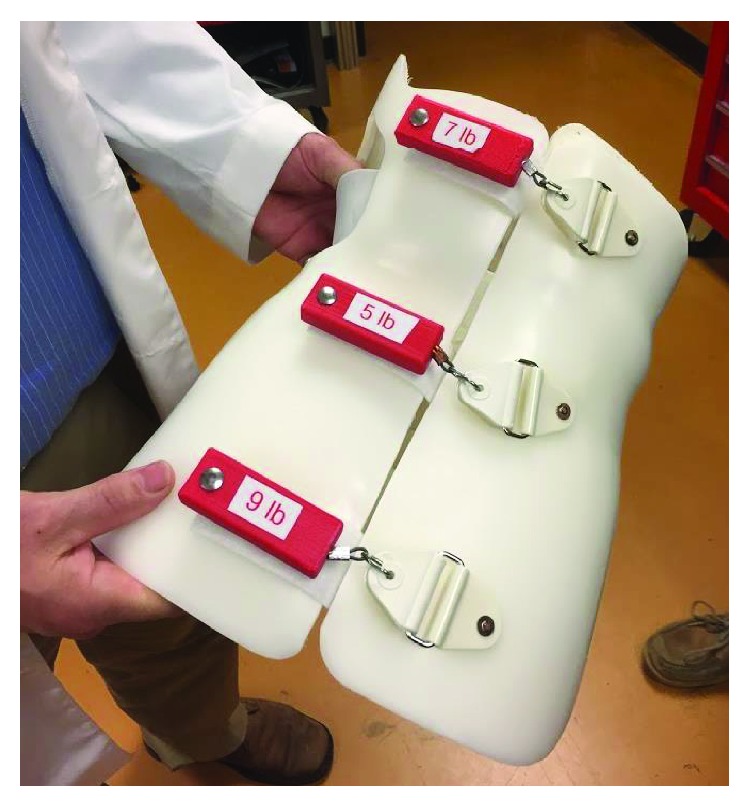
CTU devices mounted on a conventional brace in-line with the posterior fastening straps.

The conventional bracing configuration (CB) used in this study was fabricated using the Boston Brace Manual as a standard for brace customization and had three posterior fastening straps as shown in Figure 2. It started as a standard module (as all Boston braces do) and was further customized by a licensed orthotist to optimize fit and function for the unique scoliosis case [12]. The Velcro fastening straps were replaced with the CTU fastening devices as shown in Figure 3 for comparison testing of the two fastening systems.

**Figure 4:**
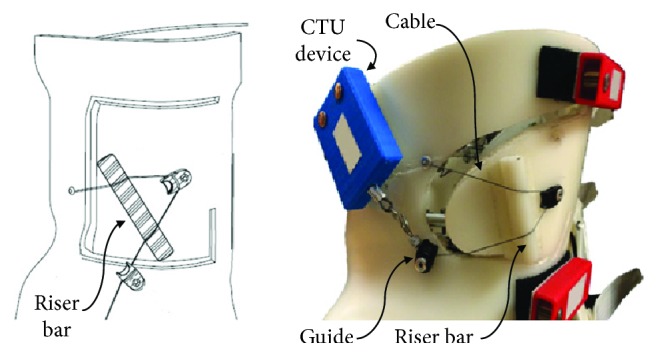
Modified brace with dynamized apical flap section. The cable from a CTU unit passed over the top of a riser bar located on the outer surface of the apical flap and anchored to the body of the brace. Tension from the CTU cable applied a force against the riser bar that pushed the flap inward.

The modified bracing (MB) configuration is shown in Figure 4 and had a dynamic cantilevered apical flap section. A cable was passed over the top surface of the apical flap that connected to a CTU device. A riser bar placed between the cable and apical pad surface transformed the tension in the cable to a force normal to the apical pad surface. The working range of the constant force spring allowed the apical flap to move inward and outward without altering the amount of force applied to the pad. A ½^″^ diameter riser bar was used, which was considered an acceptable offset height for the surface of the brace, and the offset lengths between the riser bar and the cable guides were 2.5^″^ on one side and 1^″^ on the other. For CTU cable tensions of 35 N and 71 N, the inward force applied to the riser bar was 22 N and 46 N, respectively.

### 2.3. Scoliosis Analog Model and Robotic Testing Platform

**Figure 5:**
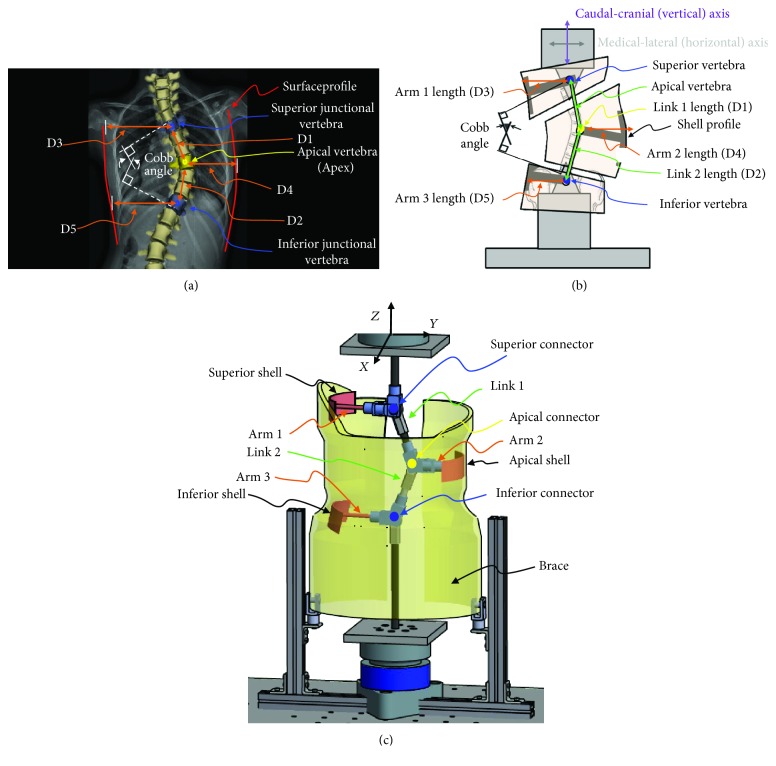
Steps in designing the scoliosis analog model. (a) Coronal plane data, (b) critical anatomy corresponding to SAM components, and (c) SAM components. Note: Example EOS scan and parameters are shown, not the actual patient scan and data used for this study.

The scoliosis analog model (SAM) [6] was a linkage-based mechanical analog model of a scoliotic spine. Characteristics and dimensions from patient records and biplanar EOS images (EOS Imaging, Paris, France) [24, 25] (IRB 14-03110-XP) were used to customize the SAM for use with a unique Boston brace. The spatial locations of the critical anatomy, including the apical vertebral body and the superior and inferior junctional vertebral bodies, corresponded to the connection points of the main linkages of the SAM (Figure 5(a)). Each of the three vertebral bodies was represented in the SAM by a linkage assembly. The distance between the critical vertebral bodies corresponded to the linkage lengths and the distance between the outer surface profile of the torso to the center of the vertebral body corresponded to the length of the arm component (Figure 5(b)). Each shell had a specific geometry that matched and interfaced with the internal contoured surface of the superior, apical, and inferior critical regions of the brace. The arm-shell components were able to pivot about the pin connector to engage with offset critical regions (Figure 5(c)). The COBB angle corresponded to the angular displacement of the linkages relative to the vertical axis. The axial rotation of the apical vertebral body was used to define the apical connector's degree of offset from the coronal (Y-Z) plane. By using these critical anatomical parameters, the experimental SAM was designed to closely replicate a clinical scoliosis deformity.

**Figure 6:**
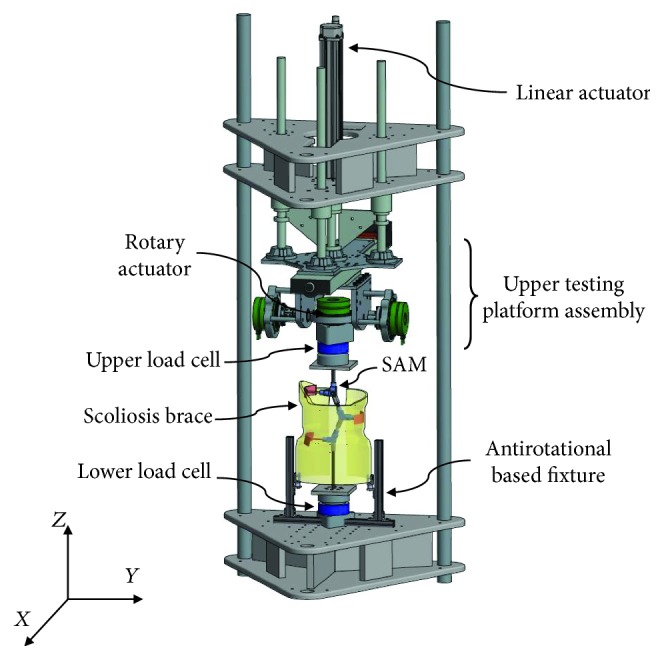
Schematic illustration of a conventional brace mounted on the scoliosis analog model located on the robotic testing platform. Downward displacement of the upper testing assembly moved the linkage arms of the SAM and simulated the angular deformity of the spine. The lower section of the brace was constrained by the test fixture to simulate proper fit of the brace on the pelvic region. Load cells located above and below the SAM recorded the multidirectional forces applied by the brace.

The overall SAM assembly was mounted into the robotic testing platform [7] as shown in Figure 6 and used to test the brace configurations. Programmed actuator movement displaced the SAM linkage system which changed the degree of frontal plane curvature (or Cobb angle) and axial rotation of the spinal deformity (Exlar linear actuator, GSX-30, Curtis-Wright; and rotary servo actuator, FHA-25C, Harmonic Drive). Two six-axis load cells placed at the superior (100M40, JR3 Inc.) and inferior (67M25S3, JR3 Inc.) ends of the SAM measured the amount of corrective spinal force applied by the brace. Throughout the simulation, the reaction forces at the upper and lower ends of the SAM were continuously recorded at 25 Hz.

### 2.4. Strap Tensiometer

**Figure 7:**
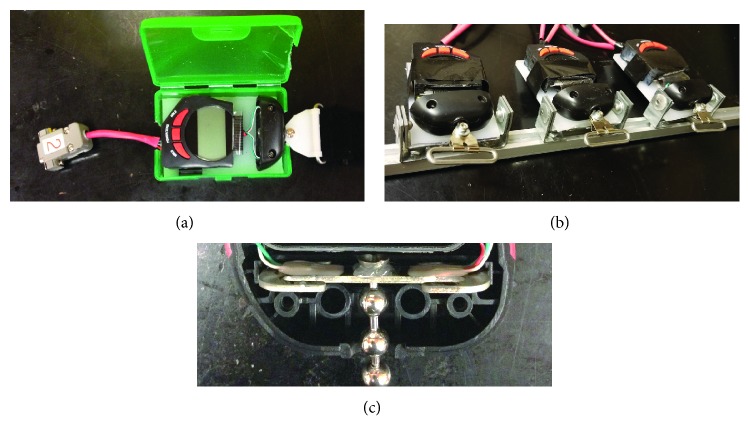
Strap tensiometer. (a) Custom tensiometer used to measure tension across the fastening straps, (b) custom fixture used for multidirectional alignment of tensiometers, and (c) strain gauge deformable plate and swivel ball chain within each tensiometer.

A digital-scale tensiometer (Berkley Digital Scales, Columbia, SC) was modified and used to measure tension in the fastening straps. The tensiometer had a maximum capacity of 222 N, 0.1 N resolution, and accuracy of 0.1% of the reading. The customized tensiometer is shown in Figure 7, and standard chafe connectors were used to attach to the fastening hardware located on the posterior aspect of the brace. To measure strap tension of a scoliosis brace, three Berkley Digital Scales were attached to the brace in line with each Velcro strap and then placed on the scoliosis analog model (SAM) [6]. The fixture that was used to rigidly attach the tensiometers to the left posterior side of the brace used a hinged connection to allow each tensiometer to pivot to be in-line with each corresponding Velcro strap on the right side (Figure 7(b)). A T-slot connector between each hinge and the vertical rail allowed for each tensiometer to be aligned with the height of each corresponding strap. The load was transferred from the Velcro strap to the deformable plate within the tensiometer at each level via a swivel ball chain connector that passed through the deformable plate and minimized any off-axis loads from being transferred to the plate (Figure 7(c)). Strain gauges installed on the deformable plate measured the amount of deformation (with accuracy of ±0.1%) that occurred when the straps were tightened. Each tensiometer was powered by a BK Precision DC power supply (3 V) and monitored using a custom National Instruments LabVIEW 2010 program.

### 2.5. Brace Testing

To meet the objectives of this study, two separate case studies were used with the scoliosis analog model. For part 1, correction of 33° CA deformity to 26° was simulated using the SAM to characterize the unique conventional brace for that patient. For part 2, a separate scoliosis case was selected due to availability of multiple duplicate braces from the manufacturer. Correction of 22° CA deformity to 18° was simulated using the SAM. Two braces were evaluated for part two, the conventional brace and a modified brace (i.e., conventional brace with dynamizing flap modification).

#### 2.5.1. Part 1: Comparative Testing Protocol of CTU Fasteners and Velcro Straps on a Conventional Brace and Data Management

**Figure 8:**
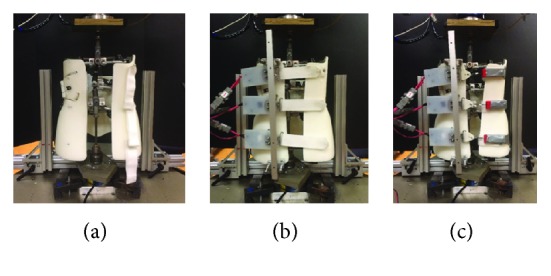
Bracing configurations. (a) No straps, (b) Velcro straps, and (c) CTU fasteners (all shown on conventional brace).

Three brace configurations shown in Figure 8 were tested to address the first objective: (1) CB with no posterior straps, (2) CB with posterior Velcro straps, and (3) CB with posterior CTU fasteners. A 20 N CTU device was used for all three straps, and the testing platform actuator was programmed to displace 10 mm at a speed of approximately 12 mm/s. Characteristics and dimensions from patient records and EOS images (EOS Imaging, Paris, France) (IRB 14-03110-XP) were used to customize the SAM for use with a custom Boston brace. The SAM was customized to represent a 13-year-old male patient with a single, right-sided thoracolumbar curve measuring 33° with less than 5° axial rotation deformity. Brace images show curve reduction to 26° and axial rotation correction to 0°.

Strap tension was recorded for each strap continuously throughout the dynamic simulation (correction of 33° CA deformity to 26° for part 1). Output from the digital tensiometers placed across the strap fasteners were assessed at the start of the simulation, at the end of the simulation (i.e., when the simulated deformity was reached), and when the SAM was reset to the initial orientation. This step was done during testing to ensure that no strap tension was lost between test runs. If a noticeable change in strap tension occurred, the straps were reset to the initial setting values and the tests were rerun.

The force component along the craniocaudal direction of the SAM represented the distractive force applied to the spine by the brace. The specific strap force contribution was derived from the dynamic force response of the two brace configurations (CB with Velcro and CB with CTU) by subtracting out the native brace (no straps) force response. The dynamic strap force contribution of the Velcro straps compared to the CTU fasteners was analyzed. Additionally, the dynamic strap tension was plotted for each strap type with respect to the amount of mediolateral displacement (i.e., brace gap separation) that occurred during the simulation. Each brace configuration was tested three times, and the data for each trial were averaged.

#### 2.5.2. Part 2: Testing Protocol of Modified Brace with Dynamized Apical Flap Section and Data Management

The second objective involved the evaluation of a dynamized apical flap section. Four brace configurations were tested: (1) CB with posterior Velcro straps, (2) CB with posterior CTU fasteners, (3) MB with posterior Velcro straps, and (4) MB with posterior CTU fasteners. Characteristics and dimensions from patient records and EOS images were used to customize the SAM for use with a second custom Boston brace. The SAM was customized to represent a 13-year-old male patient with a single, left-sided thoracolumbar curve measuring 22° with less than 5° axial rotation deformity. Brace images show curve reduction to 18° and axial rotation correction to 0°. The MB configurations were tested with three CTU load conditions acting across the apical flap: 0 N, 35.6 N, and 71.2 N. The testing platform actuator was programmed to displace 1 mm at a speed of approximately 12 mm/s.

The distractive force measured at peak simulated spinal deformity was used for comparison between the four bracing configurations. The digital tensiometers were used to set the initial strap tension setting. Strap tension was set to 18 N, 13 N, and 31 N ± 1 N at the upper, middle, and lower straps, respectively, for all bracing conditions. This strap tension selection was based on the average values measured at each level in a CTU clinical study (IRB 16-04475-XP and were approximately 18 N, 13 N, and 31 N after the orthotist tightened the straps). Each brace configuration was tested three times, and the data for each trial were averaged.

## 3. Results

### 3.1. Part 1

**Figure 9:**
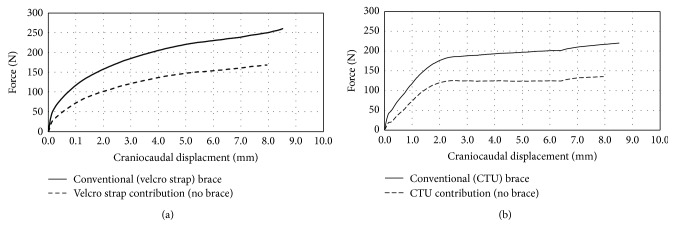
Distractive force versus vertical displacement of conventional brace with (a) Velcro strap fasteners and (b) CTU device fasteners.

Traditional Velcro fastening straps caused a continual increase in the amount of distractive force applied to the spine by the conventional brace (Figure 9(a)), while the distractive force remained fairly constant for the CTU fastener devices (Figure 9(b)). This finding supports the design rationale that the CTU fasteners can provide a more flexible dynamic brace that allows for directional movement without compromising the corrective force capacity of the brace.

**Figure 10:**
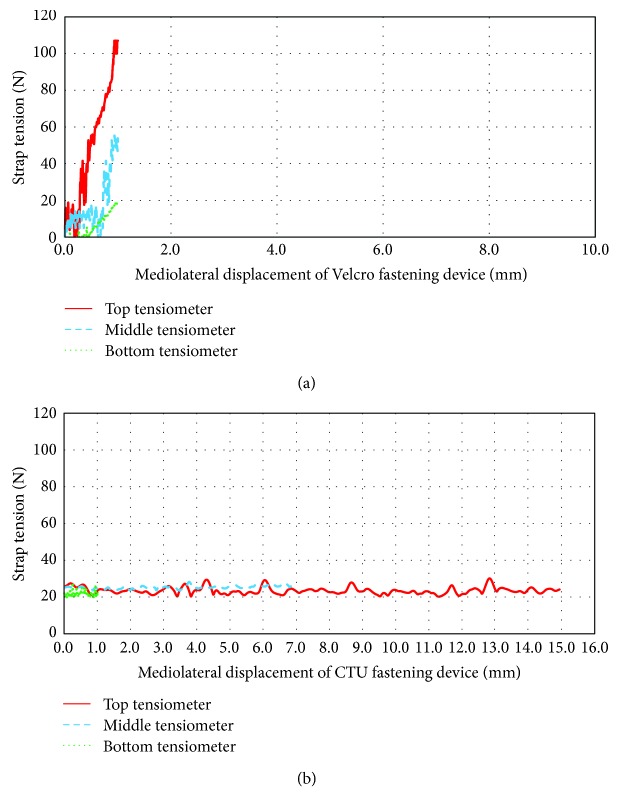
Strap tension versus mediolateral brace gap separation of conventional brace with (a) Velcro strap fasteners and (b) CTU fasteners.

Tension across the three Velcro straps of the CB configuration increased rapidly (up to 100 N) within the first mm of brace gap separation as shown in Figure 10(a) and was greatest at the upper strap. However, strap tension across all three CTU fasteners on the CB configuration remained constant at the 20 N setting and allowed up to 15 mm of brace gap separation (Figure 10(b)). Having the strap tension setting remain constant while allowing the brace to open or close across the backside of the brace allows for opportunities of deep breathing and forward bending without increased brace loads and potential user discomfort.

### 3.2. Part 2

**Figure 11:**
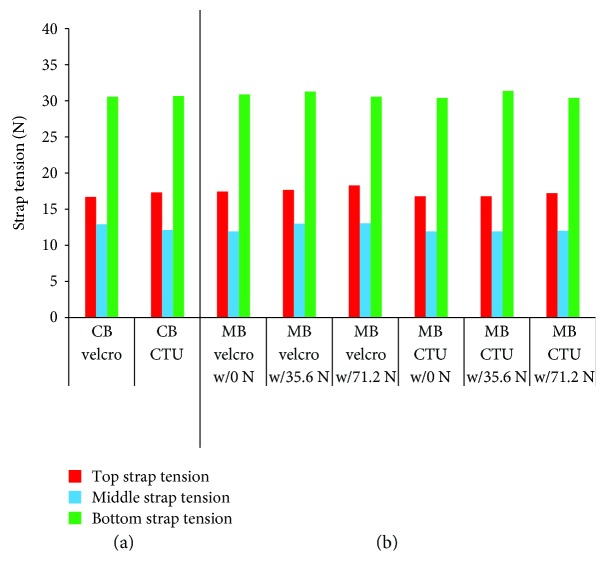
Strap tension values for different brace configurations: (a) conventional brace (Velcro versus CTU fasteners and (b) modified brace (Velcro versus CTU fasteners).

**Figure 12:**
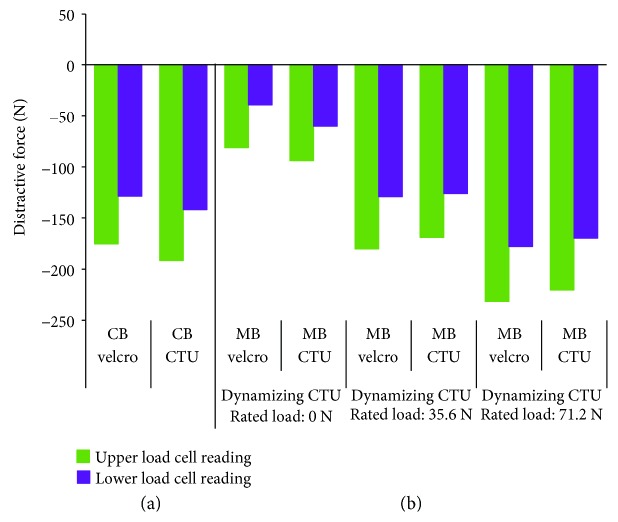
Effects of apical flap load on distractive force. (a) Conventional brace (no apical flap) and (b) modified brace with increasing apical flap loads.

For all CTU brace configurations, the CTU strap fastening tension stayed within ±0.9 N of the prescribed Velcro strap tension values (Figure 11) for brace configurations. The CTU fasteners also provided the same corrective loads (Fz) as Velcro straps when tensioned to the same level of strap tension as the Velcro straps in the conventional brace configuration (Figure 12(a)). The other force components (Fx and Fy) and the total moment values (Mt) transferred to the SAM (i.e., at the top and bottom load cells) are listed shown in Table 1 and remained minimal relative to the distractive component.

An apical flap was added to the conventional brace to create the modified brace. The apical flap alone (with no dynamized CTU input) caused a reduction in the amount of distractive force that was independent of the type of posterior fastening device used (Figure 12(b)). When the force output of a CTU device was applied across the apical flap, an increase in the distractive forces applied to the spine increased significantly at both ends of the SAM (Figure 12(b)). Further, increasing the CTU tension across the flap from 35.6 N to 71.2 N resulted in a further increase in the amount of distractive forces applied to the spine.

## 4. Discussion

This study is not without limitations. Two different scoliosis case studies were used to analyze their respective custom Boston braces in the two parts of the study (i.e., a conventional Boston brace for part 1 and a separate conventional Boston brace and modified Boston brace for part 2). However, the data sets were not mixed or used to compare to one another. The part 1 data is shown in Figures 9 and 10, and part 2 data is shown in Figures 11 and 12 and Table 1. Other brace types should be evaluated to determine if the response in how the apical flap loads affect the distractive force properties of the brace is similar. The SAM was unable to simulate the effects of soft tissue or the compliancy of the rib cage and currently cannot simulate either a double curve or a compensatory curve deformity. A single deformity curve was simulated as it was considered more prevalent in the practice of our clinical collaborators. Another limitation of this study was how tall the riser bar could be on the modified brace as well as the placement of the cable guides. For the ½^″^ diameter riser bar and flap cut-out section used in this study, an inward force of 45 to 50% of the CTU tension value would be applied.

In a study by Mac-Thiong et al. [3], mini load cells were used to measure the tension in the fastening straps of a scoliosis brace. The straps were then tightened to 20, 40, and 60 N, and the applied pressures between the torso and the brace were measured by with a force-sensing pressure pad outfitted with 192 force sensing transducers. The authors concluded that the applied pressure increased with increasing strap tension [3]. They also stated that discomfort increased with increasing strap tension and pad pressure [3]. Wong et al. found strap tension increased during deep breathing [2]. Other studies have also shown an increase in strap tension during deep breathing [5, 15–16] and have found that this led to discomfort [17]. In the current study, the posterior CTU fasteners allowed larger amounts of brace gap opening (comparable to deep breathing) without affecting the tension across the fastening straps. Use of the constant force spring within the CTU fasteners also avoids problems of strap tension loss associated with Velcro. The long-term benefit of decreasing the structural stiffness of the brace (by allowing it to open at the back side) without causing a reduction in strap fastener tension should make for a more comfortable brace to wear and improve user compliancy.

Translation of these findings to clinical practice could result in improved control of curve correction through the calculated installation of posterior fasteners and apical pad flaps during brace fabrication and prescription of strap tension levels during brace fitting. In their manual for brace fabrication, Boston Brace International recommends placing a pad on the interior surface of the brace to “provide superior medial lift to the ribs under the apex” for thoracic curves [12]. Recent studies using the SAM to study scoliosis braces have confirmed that the brace forces are multidirectional, inward and upward, at the apical level [6, 13]. The apical pad is located laterally between the rib corresponding to the apical vertebra (located slightly below the apical level due to rib cage deformation) and the iliac crest (centered about the L2/L3 disc space). The thickness of the pad is tapered such that it is thicker on the bottom and thinner on the top. Except to say that a strap should be placed at the iliac crest level and another strap placed at the level of the posterior superior iliac spine, there is no instruction on where to place the Velcro strap fasteners relative to the curve apex or how tightly each strap should be fastened. Equipped with the knowledge gained from this study, orthotists can locate and set posterior fasteners to direct the brace force vector to meet the needs of each patient's specific curve geometry. For example, a single thoracic curve could benefit from a greater distractive brace force to prevent in-brace worsening and progression of the deformity to a double curve with a compensatory lumbar curve. This could be achieved by using a calculated level of closure at each of the strap levels with prescribed strap tension levels. Specifically, for a three-strap brace, the most closure would be at the bottom strap located at the hips (i.e., the pelvis serves as fixed base for brace), medium closure at the middle strap located below the apex (i.e., for desired superior medial displacement of the apex), and least closure at the top strap located above the apex (i.e., allowing for the spine to shift and correct upwards/caudally).

## 5. Conclusions

The CTU fasteners were able to apply comparable levels of tension across the backside of a conventional brace as standard Velcro strap fasteners and may be used as an alternative to conventional Velcro straps. Additionally, the CTU fasteners allowed the back side of the brace to open or close without altering the strap tension eliminating the problem of strap tension loss. This action should improve brace comfort and increase brace wear time. Dynamizing the apical flap by using the CTU devices to apply a constant load normal to the flap surface further provided a way to easily increase the amount of distractive force applied to the spine by the brace. The next steps for this research work are to confirm radiographically that no loss in the spinal curve correction occurs when CTU fasteners are used to apply comparable amounts of strap fastening tension as traditional Velcro straps and to clinically assess if the CTU fasteners are able to improve brace comfort by allowing the backside of the brace to open or close during movements of daily living.

## Figures and Tables

**Table 1:** Corrective forces and moments applied by brace to the SAM.

Brace configuration			Upper load cell readings				Lower load cell readings			
Brace	Posterior straps	Dynamic load	F_x_ (N)	F_y_ (N)	F_z_ (N)	M_t_ (Nm)	F_x_ (N)	F_y_ (N)	F_z_ (N)	M_t_ (Nm)
CB	Velcro	N/A	−15.1	0.4	−175.4	−0.7	11.2	20.1	−128.7	0.1
CB	CTU	N/A	−16.0	1.0	−191.6	−0.5	14.8	18.3	−141.8	0.1
MB	Velcro	0 N	−12.1	−8.7	−81.2	−1.3	6.1	7.8	−39.4	0.1
MB	Velcro	35.6 N	−10.8	−7.4	−93.9	−0.7	5.1	11.4	−60.2	0.1
MB	Velcro	71.2 N	−13.5	4.5	−180.3	−1.1	9.0	25.1	−129.2	0.1
MB	CTU	0 N	−14.6	3.5	−169.0	−0.6	11.6	23.2	−126.1	0.1
MB	CTU	35.6 N	−15.2	10.0	−231.6	−0.6	11.9	33.8	−177.9	0.1
MB	CTU	71.2 N	−15.7	11.2	−220.4	−0.4	13.7	31.9	−169.7	0.1

## Data Availability

Data within this study is archived in the Biorobotics Laboratory in the Department of Orthopaedic Surgery and Biomedical Engineering at the University of Tennessee Health Science Center. Contact corresponding author for inquiries.
